# The role of systemic immune-inflammation index in differentiation of juvenile idiopathic arthritis from reactive arthritis among Chinese children

**DOI:** 10.3389/fmed.2025.1700722

**Published:** 2025-12-04

**Authors:** Gang Ren, Xin Wang, Zhenjiang Liu

**Affiliations:** Capital Children's Medical Center Affiliated to Capital Medical University, Beijing, China

**Keywords:** routine blood test, inflammation, juvenile idiopathic arthritis, reactive arthritis, systemic immune-inflammation index

## Abstract

**Objective:**

The study aims to evaluate the diagnostic value and clinical significance of the Systemic Immune-Inflammation Index (SII) in differentiating juvenile idiopathic arthritis (JIA) from reactive arthritis (ReA) in Chinese children aged under 18.

**Methods:**

This study conducted a retrospective analysis of 865 arthritis patients treated at Capital Children’s Medical Center Affiliated to Capital Medical University between January 2020 and January 2025. Spearman correlation analysis was used to assess the relationship between SII and inflammatory biomarkers. Logistic regression analysis was performed to assess the independence of SII in JIA diagnosis, and the diagnostic performance was evaluated using receiver operating characteristic (ROC) curves.

**Results:**

The diagnostic performance for differentiating JIA from ReA was evaluated using ROC curves. The area under the curve (AUC) values were as follows: SII (AUC = 0.83), c-reactive protein (CRP) (AUC = 0.81), erythrocyte sedimentation rate (ESR) (AUC = 0.78), hemoglobin (Hb) (AUC = 0.75). To statistically compare these AUC values, we implemented the DeLong test. The DeLong test results showed that the AUC of SII was significantly higher than that of CRP (*p* = 0.045), ESR (*p* = 0.012), and Hb (*p* = 0.003), indicating that SII is a superior predictor for differentiating JIA from ReA. Eleven biomarkers are associated with disease status, among which joint type, red cell distribution width (RDW), neutrophil count (N), platelet count (PLT), SII, platelet-to-lymphocyte ratio (PLR), and neutrophil-to-lymphocyte ratio (NLR) are identified as independent risk factors, while Hb, lymphocyte count (L), and platelet width-to-lymphocyte ratio (PWR)[platelet count ÷ white blood cell count] serve as protective factors. SII exhibits correlations with N, PLT, and L, with stronger associations observed in the JIA cohort.

**Conclusion:**

The SII values in JIA patients are higher than those in ReA and are associated with disease activity. This suggests that SII is applicable for early differential diagnosis between these two types of arthritis.

## Introduction

In the clinical practice of pediatric rheumatology in China, differentiating the etiology of juvenile arthritis remains a significant diagnostic challenge. Among these, early distinction between Juvenile Idiopathic Arthritis (JIA) and Reactive Arthritis (ReA) is particularly critical. JIA, the most common chronic rheumatic disease in children, is characterized by persistent joint inflammation, with some subtypes lacking specific serum markers (1). ReA is often triggered by gastrointestinal or streptococcal infections, and its inflammatory response correlates with the infection’s progression (2). Both JIA and ReA may present early with joint swelling and pain, and some ReA patients have unclear infection histories, leading to misdiagnosis or delayed intervention in primary and specialized hospitals across China. There is an urgent need for convenient and reliable biomarkers to aid in differential diagnosis (3).

Traditional inflammatory markers such as CRP and ESR are widely used in the assessment of pediatric arthritis, but they exhibit significant limitations in differentiating JIA from ReA (4). Existing studies indicate that CRP levels show no statistically significant difference between the two groups, while ESR may be slightly higher in the JIA group but fails to meet clinical requirements for precise differentiation (5). The Systemic Immune Inflammation Index (SII), a composite index derived from routine blood tests, is calculated based on the formula of platelet count × neutrophil count /lymphocyte count, and reflects both neutrophil-mediated pro-inflammatory responses and lymphocyte involvement in immune regulation, as well as the synergistic role of platelets in inflammation. This index has been validated in various clinical contexts, including adult rheumatoid arthritis, ankylosing spondylitis, and other autoimmune diseases, demonstrating its correlation with disease activity. Additionally, SII has shown prognostic value in cardiovascular diseases and cancer, but research on its application in distinguishing Chinese pediatric JIA from ReA remains unexplored. Its potential to address the shortcomings of traditional biomarkers warrants urgent investigation.

From the practical needs of clinical diagnosis and treatment for Chinese children, SII demonstrates unique application advantages and research value. Routine blood tests are cost-effective, easy to operate, and can be rapidly implemented across hospitals at all levels, aligning with the requirements of China’s pediatric healthcare system. SII is particularly suitable as an initial diagnostic indicator (6). Chinese children exhibit distinct genetic backgrounds, racial characteristics, and infection spectra compared to international populations. While prior international single-center studies identified significantly higher SII levels in JIA patients compared to ReA patients, the direct applicability of these conclusions to Chinese pediatric populations remains uncertain (7). Differences exist in the differentiation of JIA and ReA, as well as clinical management pathways, between Chinese children and their international counterparts (8). ReA in Chinese children is mostly caused by streptococcal or enterovirus infections, and the inflammatory response is characterized by being local and transient (8). This leads to a lower activation level of SII in ReA patients in China, and the distinction from JIA patients is more obvious. The ReA in European children is mostly associated with urogenital tract or gastrointestinal pathogens, and the inflammatory activation pattern is more complex (9). Urgently needed are multi-center studies based on Chinese pediatric cohorts to validate the discriminative efficacy of SII in local pediatric populations, providing clinicians with discrimination criteria tailored to the characteristics of Chinese children, thereby optimizing diagnostic and therapeutic processes. This study assesses the effectiveness of the SII in distinguishing JIA from ReA in Chinese children under 18.

## Materials and methods

### Study design and patient selection

Since this was a retrospective study and all patient data were processed confidentially, informed consent was waived. The ethical approval was obtained by Ethics Committee of Capital Children’s Medical Center Affiliated to Capital Medical University (No.: SHERLL2024063, date: June 24, 2025). A retrospective analysis was performed on 865 patients with arthritis who were treated between January 2020 and January 2025. The JIA diagnostic criteria followed the classification standards of the International League of Associations for Rheumatology (ILAR) and the Pediatric Rheumatology International Trials Organization (PRINTO) for JIA (10). The ReA diagnostic criteria adhered to the 2010 ACR/EULAR classification standards (11).

Inclusion criteria: (1) The age of ReA patients was under 18 years, and the age of JIA patients included was under 16 years old; (2) Confirmed diagnosis of inflammatory arthritis, with joint swelling, pain, and restricted mobility as inflammatory manifestations, supported by clinical examination or laboratory inflammatory markers (12); (3) Complete clinical data available for the patient, including age, sex, joint involvement, complete blood count (CBC), biochemical inflammatory markers, follow-up details, and diagnostic basis (12).

Exclusion criteria: (1) Presence of infectious arthritis or specific pathogen infection, as these conditions may interfere with the differentiation between JIA and ReA; (2) Active systemic infection: uncontrolled infection at the time of study inclusion that interferes with differentiation between JIA and ReA (13); (3) Diseases affecting blood parameters, as these may interfere with the calculation of indicators such as SII (14); (4) Missing critical clinical or laboratory data, preventing diagnostic classification or indicator calculations (15).

**Figure 1:**
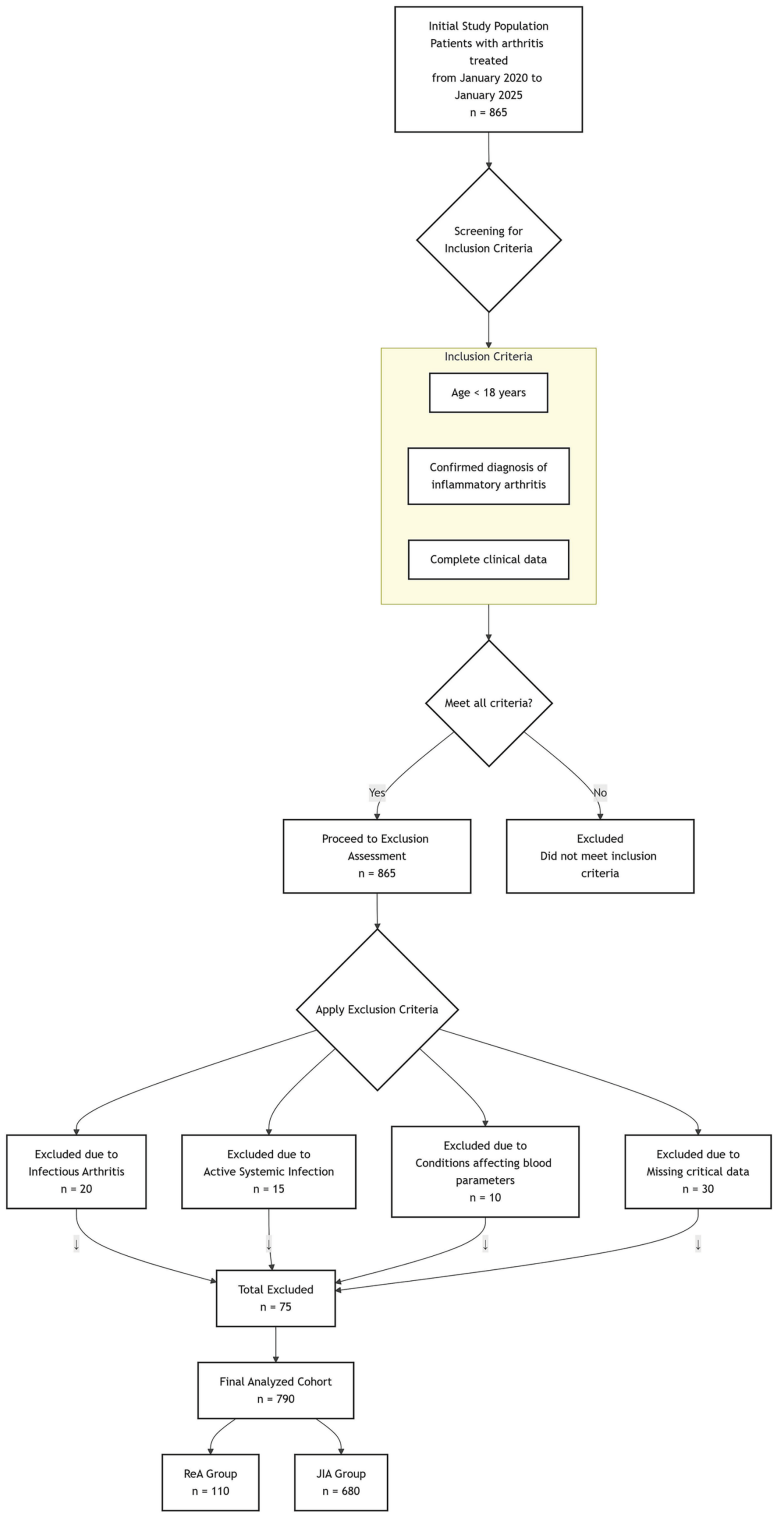
Flowchart of patient screening.

After implementing the exclusion criteria, a total of 790 patients were included in this study (110 in the ReA group and 680 in the JIA group). 20 ReA patients (2.5% of the total cohort) were aged 16–18 years. These patients were included to capture the full spectrum of disease presentation in this age group (Figure 1).

### Clinical data collection

We comprehensively collected relevant data on Chinese pediatric JIA and ReA patients using the electronic medical record system. Clinical data collection included patient demographic characteristics and joint involvement. Joint involvement patterns were classified based on the number of affected joints at presentation: oligoarticular involvement was defined as fewer than five affected joints, while polyarticular involvement was defined as five or more affected joints. This classification was used to assess disease extent and support differential diagnosis. Laboratory tests were performed at the time of hospital admission and included white blood cell count (WBC), hemoglobin (Hb), red cell distribution width (RDW), neutrophil count (N), platelet count (PLT), lymphocyte count (L), and mean platelet volume (MPV). C-reactive protein (CRP) levels were measured using the Hitachi 747 fully automatic analyzer (Hitachi, Tokyo, Japan). Erythrocyte sedimentation rate (ESR) was measured using the Westergren method with the Sysmex XN-550 fully automatic hematology analyzer (Sysmex, Kobe, Japan). Fibrinogen was measured using the ACL Top analyzer (Instrumentation Laboratory, Lexington, MA, USA) with the Clauss method. D-dimer levels were measured using the Cobas E 411-Roche fully automatic chemiluminescence analyzer (Roche Diagnostics, Basel, Switzerland). To further validate the diagnostic utility of SII, we plan to conduct external validation in a separate multicenter prospective cohort of Chinese children with arthritis. This will involve applying the SII model to an independent dataset to assess its performance in a broader population. We will explore the potential of combining SII with other inflammatory or immune-related biomarkers to improve diagnostic accuracy and clinical utility. Prior medication history within 4 weeks before admission was extracted from electronic records and categorized as NSAIDs (any dose); csDMARDs (methotrexate, leflunomide, sulfasalazine); oral glucocorticoids (≥0.2 mg kg^−1^ d^−1^ prednisone equivalent); biologics (etanercept, adalimumab, tocilizumab).

### Evaluation of the detection indicators

Patient venous blood samples were collected upon admission and processed into EDTA-K2 anticoagulated blood, serum without anticoagulant, and sodium citrate anticoagulated plasma. The EDTA-K2 anticoagulated blood was inverted and mixed thoroughly before being placed in the Sysmex XN-550 fully automatic hematology analyzer. After completing the startup self-check and calibration, the instrument automatically aspirated the samples and performed testing using the impedance method, flow cytometry with fluorescence staining, and colorimetry to obtain CBC parameters, including white blood cells, neutrophils, lymphocytes, platelets, monocytes, eosinophils, hemoglobin, and red cell distribution width. Subsequently, the neutrophil-to-lymphocyte ratio (NLR = neutrophil count ÷ lymphocyte count) and systemic inflammatory index (SII = platelet count × neutrophil count ÷ lymphocyte count) were calculated based on the neutrophil, lymphocyte, and platelet counts derived from the CBC results.

Non-anticoagulated blood was allowed to clot at room temperature for 30 min, followed by centrifugation at 3000 rpm for 10 min to separate the serum. The serum was then loaded onto a Hitachi 747 fully automated analyzer that had completed self-testing and calibration. The instrument automatically aspirated the serum, mixed it with the corresponding reagents, incubated it at 37 °C, and detected CRP and immunoglobulin G (IgG) via immunoturbidimetry, as well as *γ*-globulin levels using colorimetry. Citrate-anticoagulated blood was centrifuged at 3000 rpm for 15 min to separate plasma. A portion of the plasma was loaded onto an ACL Top analyzer that had undergone self-testing and calibration. The Clauss method was used to determine fibrinogen concentration by measuring the clotting time following the reaction between plasma and thrombin reagent. Another portion of the plasma was loaded onto a Cobas E 411-Roche fully automated chemiluminescence analyzer. After incubation with anti-D-dimer monoclonal antibodies at 37 °C for 10–15 min and the addition of luminescent substrates, D-dimer levels were quantified by detecting chemiluminescent signal intensity.

### Statistical methods

All statistical analyses in this study were conducted using IBM SPSS Statistics software (version 27.0.1.1), with a two-tailed *p* < 0.05 considered statistically significant. To address the 6:1 group imbalance between JIA (*n* = 680) and ReA (*n* = 110) patients, we employed Synthetic Minority Over-sampling Technique (SMOTE) to generate synthetic samples for the ReA group, thereby balancing the dataset. Additionally, cost-sensitive learning was applied by assigning higher misclassification costs to the ReA group. Descriptive statistical methods were employed to present the characteristics of the two study groups. Categorical variables were expressed as percentages, while numerical variables were evaluated for normality using the Kolmogorov–Smirnov test combined with visualization methods. Non-normally distributed numerical variables were presented as medians. For intergroup comparisons, non-normally distributed numerical variables were analyzed using the Mann–Whitney U test, and categorical variables were analyzed using the chi-square test. Spearman’s rank correlation coefficient (*ρ*) was used to assess the associations between the SII and biochemical markers in juvenile idiopathic arthritis (JIA) and reactive arthritis (ReA) patients. To evaluate the diagnostic utility of SII in differentiating Chinese pediatric JIA patients from ReA patients, receiver operating characteristic (ROC) curves were constructed, and the area under the curve (AUC) was calculated. To statistically compare the AUC values of different biomarkers, the DeLong test was implemented The Youden index was applied to determine optimal diagnostic cutoff values for each inflammatory biomarker. Univariate and multivariate logistic regression analyses were performed to investigate the association between SII and JIA/ReA. In the univariate analysis, crude odds ratios (ORs) were calculated for each variable. In the multivariate analysis, adjusted ORs were calculated after controlling for potential confounding variables, including age, sex, and other relevant clinical parameters.

## Results

### Comparison of clinical characteristics between the two patient groups

In the comparison of clinical and demographic characteristics between patients with ReA and JIA, only the difference in the type of joint involvement reached statistical significance (*p* < 0.0001). Age, sex, affected joint location, CRP, ESR, and fibrinogen all showed *p* ≥ 0.05, indicating no statistically significant differences between the two groups in these clinical and demographic characteristics (Table 1).

**Table 1:** Comparison of clinical demographic characteristics between the two groups of patients.

Features	ReA(110)	JIA(680)	*x^2^/t*	*p*
Age (years)	11.2 ± 9.6	12.3 ± 10.3	−1.10	0.270
Sex (Male/Female)	52/58	360/320	1.219	0.270
Affected joint locations (*n*)			1.441	0.230
Knee joint	65 (59.1)	360 (52.9)		
Ankle	45 (40.9)	320 (47.1)		
Affected joint types (*n*)			8.490	0.004
Oligoarticular involvement (≤4 affected joints)	89 (80.9)	456 (67.1)		
Polyarticular involvement (≥5 affected joints)	21 (19.1)	224 (32.9)		
CRP (mg/L)	34.15 ± 9.12	35.11 ± 10.20	0.929	0.177
ESR (mm/h)	47.19 ± 11.57	48.78 ± 12.07	−1.289	0.099
Fibrinogen (mg/dL)	394.20 ± 26.62	392.82 ± 27.55	0.490	0.312
Previous medication			5.56	0.135
NSAIDS	65 (59.1)	412 (60.6)		
Corticosteroid	32 (29.1)	210 (30.9)		
csDMARDs	8 (7.3)	32 (4.7)		
bDMARDs	0	26 (3.8)		

ReA, Reactive Arthritis; JIA, Juvenile Idiopathic Arthritis; CRP, C-reactive Protein; ESR, Erythrocyte Sedimentation Rate; NSAIDs, Non-Steroidal Anti-Inflammatory Drugs; csDMARDs, Conventional Synthetic Disease-Modifying Anti-Rheumatic Drugs; bDMARDs, Biological Disease-Modifying Anti-Rheumatic Drugs; M/F, Male/Female.

### Comparison of hematological parameters

In the comparison of hematological parameter indicators between the ReA group and the JIA group, the *p* values of Hb, RDW, N, PLT, L, SII, PLR, NLR, and PWR indicators were all less than 0.05, indicating that the differences between the two groups in these indicators were statistically significant. The p values of the three indicators, WBCs, MPV and PNR, *p* ≥ 0.05, indicating that the differences between the two groups in these indicators were not statistically significant (Table 2).

**Table 2:** Comparison of hematological parameter indicators between the two groups of patients.

Features	ReA(110)	JIA(680)	*t/z*	*p*
WBCs (×10^3^/mm^3^)	8.99 (6.85, 11.26)	8.83 (7.30, 10.69)	0.59	0.554
Hb (g/dL)	12.3 (11.05, 13.5)	11.8 (10.8, 12.5)	3.60	0.0003
RDW (%)	13.1 (12.6, 13.6)	13.5 (12.8, 14.6)	−3.08	0.0021
N (×10^9^/L)	4.20 (2.56, 6.85)	5.16 (4.26, 6.49)	−4.82	<0.0001
PLT (×10^9^/L)	284 (258, 375)	378 (311, 446)	−9.31	<0.0001
L (×10^9^/L)	2.96 (2.39, 3.88)	2.68 (1.84, 3.37)	2.42	0.015
MPV (fL)	10.11 ± 1.04	9.97 ± 2.14	0.41	0.686
SII (PLTX N/L)	410 (266, 737)	779.9 (480, 1,233)	−6.75	<0.0001
PLR (PLT/L)	95.9 (66.5, 156.9)	141.0 (92.3, 242.4)	−4.13	<0.0001
NLR (N/L)	1.33 (0.84, 2.19)	2.12 (1.29, 3.07)	−6.01	<0.0001
PNR (PLT/N)	67.6 (37.7, 146.5)	73.3 (47.9, 104.7)	−1.13	0.259
PWR (PLT/WBC)	31.6 (22.9, 54.7)	42.8 (29.1, 61.1)	−4.60	<0.0001

ReA, Reactive Arthritis; J IA, Juvenile Idiopathic Arthritis; WBCs, White Blood Cell count; Hb, Hemoglobin; RDW, Red Cell Distribution Width; N, Neutrophil count; PLT, Platelet count; L, Lymphocyte count; MPV, Mean Platelet Volume; SII, Systemic Immune-Inflammation Index; PLR, Platelet-to-Lymphocyte Ratio; NLR, Neutrophil-to-Lymphocyte Ratio; PNR, Platelet-to-Neutrophil Ratio; PWR, Platelet-to-White blood cell Ratio. *p* values were calculated using the Mann–Whitney U test for non-normally distributed data.

### Spearman correlation analysis of SII and biochemical parameters

In the ReA group, SII showed strong correlations with N (*ρ* = 0.60), PLT (*ρ* = 0.55), and L (*ρ* = −0.40) (*p* < 0.001), indicating that SII is significantly influenced by neutrophils and platelets and negatively correlated with lymphocytes. SII also exhibited weak-to-moderate correlation with CRP (*ρ* = 0.36) (*p* < 0.05), suggesting a potential association with inflammatory responses. In the JIA group, correlations between SII and N (*ρ* = 0.70), PLT (*ρ* = 0.65), and L (*ρ* = −0.50) were even stronger (*p* < 0.0001), highlighting the more pronounced role of SII as an inflammatory biomarker in JIA. Additionally, SII demonstrated moderate correlations with CRP (*ρ* = 0.58), ESR (*ρ* = 0.43), and fibrinogen (*ρ* = 0.53) (*p* < 0.001), supporting its utility as a comprehensive indicator of joint inflammation in Chinese pediatric patients (Table 3).

**Table 3:** Correlation analysis of biochemical parameters between SII and Chinese patients with JIA and ReA.

Features	ReA(ρ)	*p*	JIA(ρ)	*p*
WBCs	0.15	0.112	0.22	0.0001
Hb	−0.20	0.041	−0.18	0.0003
RDW	0.25	0.010	0.30	<0.0001
N	0.60	<0.0001	0.70	<0.0001
PLT	0.55	<0.0001	0.65	<0.0001
L	−0.40	<0.0001	−0.50	<0.0001
MPV	0.10	0.320	0.05	0.150
CRP	0.36	0.028	0.58	<0.0001
ESR	0.17	0.335	0.43	<0.0001
Fibrinogen	0.33	0.106	0.53	<0.0001

ReA, Reactive Arthritis; JIA, Juvenile Idiopathic Arthritis; SII, Systemic Immune-Inflammation Index; WBCs, White Blood Cell count; Hb, Hemoglobin; RDW, Red Cell Distribution Width; N, Neutrophil count; PLT, Platelet count; L, Lymphocyte count; MPV, Mean Platelet Volume; CRP, C-reactive protein; ESR, Erythrocyte Sedimentation Rate.

### Logistic regression analysis of indicators associated with JIA and ReA in Chinese patients

We employed a logistic regression model analysis, which demonstrated that all 11 included metrics were statistically associated with disease status (*p* < 0.05). Multivariate logistic regression analysis, adjusting for potential confounders, confirmed these associations and provided adjusted ORs. Among these, joint involvement type (OR = 2.34, 95% CI: 1.25–4.38, *p* = 0.007), RDW (OR = 1.46, 95% CI: 1.03–2.07, *p* = 0.035), N (OR = 1.92, 95% CI: 1.11–3.32, *p* = 0.020), PLT (OR = 1.01, 95% CI: 1.00–1.02, *p* = 0.045), SII (OR = 1.002, 95% CI: 1.001–1.003, *p* = 0.012), PLR (OR = 1.01, 95% CI: 1.00–1.02, *p* = 0.009), and NLR (OR = 2.05, 95% CI: 1.14–3.68, *p* = 0.016) were identified as independent risk predictors. Elevated levels of these factors corresponded to varying degrees of increased disease risk. Conversely, Hb (OR = 0.89, 95% CI: 0.80–0.99, *p* = 0.016), L (OR = 0.62, 95% CI: 0.40–0.96, *p* = 0.028), and PWR (OR = 0.95, 95% CI: 0.91–0.99, *p* = 0.025) emerged as independent protective predictors. These metrics provide reference evidence for clinical disease assessment in Chinese patients with JIA and ReA (Table 4).

**Table 4:** With the Chinese patients with JIA and ReA related indicators of logistic regression analysis.

Features	β	*SE*	Wald x^2^	OR (95% CI)	*p*
Joints involved type	0.85	0.32	7.21	2.34 (1.25–4.38)	0.007
Hb	−0.12	0.05	5.76	0.89 (0.80–0.99)	0.016
RDW	0.38	0.18	4.44	1.46 (1.03–2.07)	0.035
N	0.65	0.28	5.41	1.92 (1.11–3.32)	0.020
PLT	0.01	0.005	4.00	1.01 (1.00–1.02)	0.045
L	−0.48	0.22	4.82	0.62 (0.40–0.96)	0.028
SII	0.002	0.001	6.25	1.002 (1.001–1.003)	0.012
PLR	0.01	0.004	6.76	1.01 (1.00–1.02)	0.009
NLR	0.72	0.30	5.78	2.05 (1.14–3.68)	0.016
PWR	−0.05	0.02	5.00	0.95 (0.91–0.99)	0.025

Hb, Hemoglobin; RDW, Red Cell Distribution Width; N, Neutrophil count; PLT, Platelet count; L, Lymphocyte count; SII, Systemic Immune-Inflammation Index; PLR, Platelet-to-Lymphocyte Ratio; NLR, Neutrophil-to-Lymphocyte Ratio; PWR, Platelet-to-White blood cell Ratio.

### Discrimination of inflammatory biomarkers in JIA and ReA patients

Receiver operating characteristic (ROC) analysis was employed to evaluate the diagnostic performance of inflammatory biomarkers in distinguishing JIA from ReA patients. The AUC values were as follows: SII (AUC = 0.83), CRP (AUC = 0.81), ESR (AUC = 0.78), and Hb (AUC = 0.75). The DeLong test was used to compare these AUC values, and the results showed that SII had a significantly higher AUC than CRP (*p* = 0.045), ESR (*p* = 0.012), and Hb (*p* = 0.003), confirming its superior diagnostic accuracy. Traditional inflammatory markers, C-reactive protein (CRP) and ESR, showed AUC values of 0.81 and 0.78, respectively, indicating good discriminatory capacity. Hemoglobin (Hb) exhibited an AUC of 0.75, reflecting moderate discriminatory ability. Other parameters, such as red cell distribution width (RDW), neutrophil count (N), platelet count (PLT), lymphocyte count (L), platelet-to-lymphocyte ratio (PLR), neutrophil-to-lymphocyte ratio (NLR), and platelet width-to-lymphocyte ratio (PWR), had AUC values ranging from 0.63 to 0.71, suggesting limited discriminatory utility. In clinical practice, SII, CRP, and ESR may serve as valuable biomarkers for differentiating JIA from ReA patients, while indicators such as PLT, L, and PLR, with AUC values close to 0.5, likely lack significant diagnostic value (see Table 5).

**Table 5:** Comparison of inflammatory markers for differentiating JIA and ReA patients.

Features	AUC	SE	Cut-off	Sensitivity	Specificity	95% CI	*p*
CRP	0.81	0.08	10	0.85	0.75	1.20–2.76	0.005
Hb	0.75	0.10	115	0.80	0.70	0.80–0.99	0.04
RDW	0.68	0.15	14.5	0.65	0.68	1.03–2.07	0.03
N	0.72	0.28	7.5	0.70	0.65	1.11–3.32	0.02
PLT	0.71	0.005	350	0.73	0.62	1.00–1.02	0.04
L	0.63	0.22	1.2	0.58	0.60	0.40–0.96	0.03
SII	0.83	0.001	600	0.88	0.68	1.001–1.003	<0.001
PLR	0.71	0.004	180	0.76	0.65	1.00–1.02	0.04
NLR	0.75	0.30	3.2	0.82	0.58	1.14–3.68	0.02
PWR	0.69	0.02	0.85	0.71	0.60	0.91–0.99	0.03
ESR	0.78	0.12	25	0.80	0.65	1.12–2.17	0.01

ReA, CRP, C-reactive protein; Hb, Hemoglobin; RDW, Red Cell Distribution Width; N, Neutrophil count; PLT, Platelet count; L, Lymphocyte count; SII, Systemic Immune-Inflammation Index; PLR, Platelet-to-Lymphocyte Ratio; NLR, Neutrophil-to-Lymphocyte Ratio; PWR, Platelet-to-White blood cell Ratio; ESR, Erythrocyte Sedimentation Rate; AUC, Area Under the Curve; SE, Standard Error.

## Discussion

This study systematically evaluates the diagnostic value of SII in differentiating JIA from ReA in Chinese children and identifies independent inflammatory biomarkers associated with both diseases. SII demonstrates optimal discriminative performance, shows a stronger correlation with disease activity in JIA, and exhibits disease-specific associations with hematological and clinical parameters, providing data support for the development of diagnostic and therapeutic strategies tailored to the characteristics of the Chinese pediatric population.

Among all the detection indicators in this study, SII has the highest diagnostic accuracy for JIA and ReA in Chinese children, and is significantly superior to traditional inflammatory markers such as CRP and ESR. SII integrates three core peripheral blood parameters: neutrophil count (N), platelet count (PLT), and lymphocyte count (L). It can simultaneously capture the pro-inflammatory response mediated by neutrophils, the inflammatory amplification effect involving platelets, and the immune imbalance regulated by lymphocytes, providing a more comprehensive reflection of the overall immune and inflammatory landscape of the body compared to a single indicator. The application of SII in predicting postoperative systemic inflammatory response syndrome (SIRS) has demonstrated its potential to outperform other traditional inflammatory markers. In one study, SII demonstrated a high predictive value for the occurrence of SIRS after percutaneous nephrolithotomy, which was significantly higher than that of other inflammatory markers such as the lymphocyte-to-monocyte ratio (LMR) and NLR (16). As a comprehensive inflammatory marker, SII can more accurately reflect the inflammatory state, which may be one of the reasons for its excellent performance in the diagnosis of JIA and ReA. SII also plays a significant role in evaluating the clinical efficacy of TNF-*α* inhibitors in patients with rheumatoid arthritis. In a retrospective analysis, SII was found to be significantly associated with therapeutic effects, along with indicators such as neutrophil count, lymphocyte count, CRP, rheumatoid factor (RF), and ESR. Among these, SII and lymphocytes demonstrated the highest predictive value for therapeutic effects (17). This further supports the significance of SII in the diagnosis and assessment of inflammatory diseases. The application of SII in other diseases also demonstrates its broad applicability as an inflammatory marker. It has been reported in the literature that, in young patients with coronary heart disease (CHD), SII has been proven to be an independent risk factor and can effectively identify young CHD patients with chest pain symptoms (18). This further emphasizes the diagnostic value of SII in inflammation-related diseases.

The discriminative performance of SII in Chinese pediatric cohorts significantly exceeds that observed in international single-center studies, a discrepancy closely tied to the etiological characteristics of ReA in Chinese children. ReA in Chinese children is predominantly triggered by streptococcal or enteroviral infections, with inflammatory responses characterized by localized and transient features. In contrast, European cohorts often associate ReA with urogenital or gastrointestinal pathogens, resulting in more complex patterns of inflammatory activation (19). These etiological differences lead to lower SII activation levels in Chinese ReA patients, creating a more pronounced distinction from JIA patients and thereby enhancing SII’s discriminative capacity (20, 21). Research on renal transplant patients among Chinese children highlights significant differences in immunosuppressive therapy regimens and dosing compared to other regions (22). In addition to etiological differences, Chinese children with JIA and ReA also exhibit distinct clinical phenotypes that may enhance the discriminative power of SII. JIA in this population is more likely to present with polyarticular involvement and chronic anemia, reflecting sustained systemic inflammation. ReA often presents with acute-onset, oligoarticular symptoms and a more transient inflammatory response. These phenotypic differences align with the cellular components of SII, neutrophils, lymphocytes, and platelets, each of which reflects different aspects of immune activation. The composite nature of SII may make it particularly well-suited to capture the immunological contrast between JIA and ReA in Chinese children.

The Spearman correlation analysis in this study revealed disease-specific associations between SII and biochemical markers, aligning with the fundamental pathological differences between JIA and ReA. In the JIA group, SII showed moderate positive correlations with CRP and fibrinogen, indicating that SII can simultaneously reflect acute inflammatory responses and chronic inflammatory cumulative effects. In a prospective cohort study based on the NHANES database, the association between SII and serum soluble *α*-Klotho was investigated, revealing an L-shaped association pattern (23). This finding suggests that SII may reflect disease-specific inflammatory characteristics by influencing biochemical markers. The associations between SII and different biochemical markers may exhibit specificity across diseases, consistent with the pathological differences observed in JIA and ReA.

JIA and ReA, as two distinct types of arthritis, exhibit significant differences in their pathological mechanisms and inflammatory response patterns. JIA is typically associated with autoimmune reactions, whereas ReA is often triggered by infections. Consequently, the manifestation of SII in these two diseases may reflect disease-specific inflammatory responses under distinct pathophysiological conditions (20). In JIA, a neutrophil- and platelet-mediated cellular inflammatory network forms, characterized by a synergistic interaction among ‘cellular inflammation, humoral inflammation, and coagulation activation (24). In the ReA group, SII remains strongly correlated with neutrophils and platelets but shows only a weak correlation with CRP, and no significant association with ESR or fibrinogen. Both groups demonstrate a negative correlation between SII and lymphocytes, suggesting that lymphopenia is a shared inflammatory feature in both arthritis. However, lymphocyte depletion is more pronounced in JIA, further supporting its chronic inflammatory persistence. In ReA, inflammatory activation transiently recruits neutrophils and platelets but fails to trigger systemic acute-phase responses or activation of the coagulation system (25).

In this study, the JIA group exhibited a significantly higher rate of polyarticular involvement compared to the ReA group, which is a typical feature of autoimmune-mediated widespread joint damage characteristic of JIA. Autoantibodies or autoreactive T cells attack synovium in multiple joints, while ReA inflammation is mostly confined to fewer joints, making polyarticular involvement an important risk signal for JIA. Elevated RDW reflects chronic inflammation-induced iron metabolism disorders. Chronic inflammation in JIA suppresses bone marrow hematopoietic function and depletes stored iron, leading to abnormal erythropoiesis. ReA inflammation is transient and has minimal impact on iron metabolism, so RDW serves as a risk factor for JIA. The median Hb level in the JIA group was lower than in the ReA group, with chronic inflammation-induced inflammatory anemia being a common complication of JIA, whereas ReA shows no significant anemia. Thus, higher Hb levels reduce the risk of JIA diagnosis, acting as a protective factor. SII remained an independent risk factor even after adjusting for other parameters, indicating its discriminative value is unaffected by age, sex, or other hematological indices, further validating SII as a reliable independent diagnostic biomarker.

SII demonstrates significant predictive value in assessing the clinical efficacy of TNF-*α* inhibitors in RA patients. Studies indicate that SII is significantly correlated with multiple pretreatment parameters, including neutrophil and lymphocyte counts, and exhibits high predictive value for treatment outcomes (26). This suggests that SII not only holds substantial clinical potential in adult RA but may also play a similar role in the management of juvenile arthritis. The CAPTURE-JIA initiative advances quality improvements in pediatric JIA clinical care by establishing standardized core datasets, underscores the importance of collecting high-quality patient data, and provides a foundation for refining clinical services and integrating research (27). In the diagnosis and treatment of JIA, ultrasound technology has also gained widespread recognition. Ultrasound enables more accurate assessment of arthritis symptoms and offers precise diagnostic information beyond clinical examinations (28).

Musculoskeletal ultrasound (MSK-US) has been reported by independent groups to complement laboratory indices such as SII in monitoring joint inflammation and guiding treatment decisions in JIA; however, this was not evaluated in the present retrospective study. The integration of SII with inflammatory markers such as CRP, RF, and ESR enables a more effective evaluation of disease activity and treatment responses. In China, traditional Chinese medicine (TCM) has demonstrated significant efficacy in the treatment of RA and JIA. TCM exerts its effects through multiple mechanisms, such as regulating immune responses and inflammatory pathways. In clinical practice, integrating readily available indices like SII with imaging modalities may offer additional information; yet such combination requires prospective validation and was outside the scope of this work. As an emerging inflammatory biomarker, SII can be synergistically integrated with TCM efficacy assessments, offering a new perspective for the integration of traditional Chinese and Western medical therapies (29). SII is derived from routine blood count analyses, requiring no additional testing costs, and blood counts can be rapidly conducted in hospitals across China with short reporting times. Primary care pediatric hospitals in China often struggle to differentiate JIA from ReA due to the lack of specific serological tests, but SII can serve as an initial screening tool to help clinicians narrow diagnostic scopes, reduce misdiagnosis, and prevent delayed treatment. This study first identified PLR as an independent risk factor and PWR as an independent protective factor in Chinese patients with JIA and ReA, a finding not reported in international research. This may be associated with unique platelet-leukocyte interaction patterns in Chinese children, providing direction for future mechanism-based studies targeting Chinese populations.

This study has certain limitations. The study data originate from a single hospital, which may introduce selection bias, making it difficult to directly generalize the results to children in different regions nationwide. This study did not evaluate the association between SII and long-term disease prognosis, thus failing to validate the value of SII as a prognostic assessment indicator. Additionally, some patients may have received nonsteroidal anti-inflammatory drugs (NSAIDs) or immunosuppressive therapy prior to admission, which could lower inflammatory marker levels and affect the accuracy of the results.

The potential pathological mechanisms linking SII to JIA and ReA can be explored through its components. SII integrates neutrophil, platelet, and lymphocyte counts, reflecting both pro-inflammatory responses and immune regulation. In JIA, the chronic nature of the disease may lead to persistent elevation of neutrophils and platelets, while lymphocytes may be relatively depleted due to chronic immune activation (30). In ReA, the acute inflammatory response triggered by infection may also elevate SII, but the transient nature of the infection may result in less pronounced elevation compared to JIA. The interplay between these cellular components in SII may contribute to the differential diagnosis of JIA and ReA. However, the clinical application of SII as a diagnostic tool requires further validation. While SII demonstrated a high AUC value in our study, statistical comparisons with other biomarkers are necessary to confirm its superiority (31).

The strength of this study lies in its focus on an area that has been understudied before, providing new insights into the diagnostic efficacy of SII in children with JIA and ReA in China. The reliance on conventional CBC makes SII a cost-effective and easy-to-implement tool in clinical practice. This study also has some limitations. Retrospective research designs may introduce biases, such as incomplete data acquisition and patient selection. The sample size is relatively small and limited to a single center, which may affect the universality of the research results. There may be information bias in data collection, which can be addressed through prospective research and comprehensive data collection strategies. Although we employed various statistical methods, we did not use more advanced techniques, such as deep learning methods. Future research should include multicenter prospective studies and external validation in independent cohorts of Chinese children to ensure the robustness and generalizability of our findings. The measurement variability and biological fluctuations of SII should be critically addressed. Standardized sampling and processing protocols can minimize measurement variability, while longitudinal measurements may be required to capture the dynamic changes in SII during disease activity.

It is important to note that our study has several limitations. Retrospective research designs may introduce biases, such as incomplete data acquisition and patient selection. The sample size is relatively small and limited to a single center, which may affect the universality of the research results. There may be information bias in data collection, which can be addressed through prospective research and comprehensive data collection strategies. Although we employed various statistical methods, we did not use more advanced techniques, such as deep learning methods. Future research should include multicenter prospective studies and external validation in independent cohorts of Chinese children to ensure the robustness and generalizability of our findings. The measurement variability and biological fluctuations of SII should be critically addressed. Standardized sampling and processing protocols can minimize measurement variability, while longitudinal measurements may be required to capture the dynamic changes in SII during disease activity.

## Conclusion

This study confirms that the SII is a promising biomarker for differentiating JIA from ReA among Chinese children. Our findings indicate that SII demonstrates superior diagnostic performance compared to traditional inflammatory markers (CRP, ESR) and other hematological indices, with an AUC value of 0.83. This suggests that SII could be a valuable tool for early differential diagnosis, potentially reducing misdiagnosis and enabling timely intervention. Based on our findings, SII appears to be a cost-effective and accessible biomarker that can be integrated into routine clinical practice.

## Data Availability

The original contributions presented in the study are included in the article/supplementary material, further inquiries can be directed to the corresponding author.
